# SpliceAI-visual: a free online tool to improve SpliceAI splicing variant interpretation

**DOI:** 10.1186/s40246-023-00451-1

**Published:** 2023-02-10

**Authors:** Jean-Madeleine de Sainte Agathe, Mathilde Filser, Bertrand Isidor, Thomas Besnard, Paul Gueguen, Aurélien Perrin, Charles Van Goethem, Camille Verebi, Marion Masingue, John Rendu, Mireille Cossée, Anne Bergougnoux, Laurent Frobert, Julien Buratti, Élodie Lejeune, Éric Le Guern, Florence Pasquier, Fabienne Clot, Vasiliki Kalatzis, Anne-Françoise Roux, Benjamin Cogné, David Baux

**Affiliations:** 1 Département de Génétique Médicale, Groupe Hospitalier Universitaire de la Pitié Salpêtrière, AP-HP.Sorbonne Université, Laboratoire de Médecine Génomique Sorbonne Université, Paris, France; 2 Laboratoire de Biologie Médicale Multi-Sites SeqOIA (laboratoire-seqoia.fr/), Paris, France; 3 Nantes Université, CHU Nantes, Service de Génétique Médicale, 44000 Nantes, France; 4 Service de Génétique, Inserm U1253, CHRU de Tours, Tours, France; 5 Laboratoire de Génétique Moléculaire, CHU de Montpellier, Université de Montpellier, Montpellier, France; 6 Service de Médecine Génomique, Maladies de Système et d’Organe, Fédération de Génétique et de Médecine Génomique, DMU BioPhyGen, APHP Centre-Université Paris Cité, Hôpital Cochin, Paris, France; 7 Centre de référence des maladies neuromusculaires Nord/Est/Ile de France, Hôpital Pitié-Salpêtrière, APHP, Paris, France; 8 Inserm, U1216, CHU Grenoble Alpes, Grenoble Institut Neurosciences, Université Grenoble Alpes, Grenoble, France; 9 PhyMedExp, INSERM, CNRS, Université de Montpellier, Montpellier, France; 10 Centre mémoire, Inserm U1172 DistALZ, Licend, Univ Lille, CHU Lille, 59000 Lille, France; 11 INM, Univ Montpellier, INSERM, Montpellier, France; 12 INM, Univ Montpellier, INSERM, CHU Montpellier, Montpellier, France

## Abstract

**Abstract:**

SpliceAI is an open-source deep learning splicing prediction algorithm that has demonstrated in the past few years its high ability to predict splicing defects caused by DNA variations. However, its outputs present several drawbacks: (1) although the numerical values are very convenient for batch filtering, their precise interpretation can be difficult, (2) the outputs are delta scores which can sometimes mask a severe consequence, and (3) complex delins are most often not handled. We present here SpliceAI-visual, a free online tool based on the SpliceAI algorithm, and show how it complements the traditional SpliceAI analysis. First, SpliceAI-visual manipulates raw scores and not delta scores, as the latter can be misleading in certain circumstances. Second, the outcome of SpliceAI-visual is user-friendly thanks to the graphical presentation. Third, SpliceAI-visual is currently one of the only SpliceAI-derived implementations able to annotate complex variants (e.g., complex delins). We report here the benefits of using SpliceAI-visual and demonstrate its relevance in the assessment/modulation of the PVS1 classification criteria. We also show how SpliceAI-visual can elucidate several complex splicing defects taken from the literature but also from unpublished cases. SpliceAI-visual is available as a Google Colab notebook and has also been fully integrated in a free online variant interpretation tool, MobiDetails (https://mobidetails.iurc.montp.inserm.fr/MD).

**Graphical abstract:**

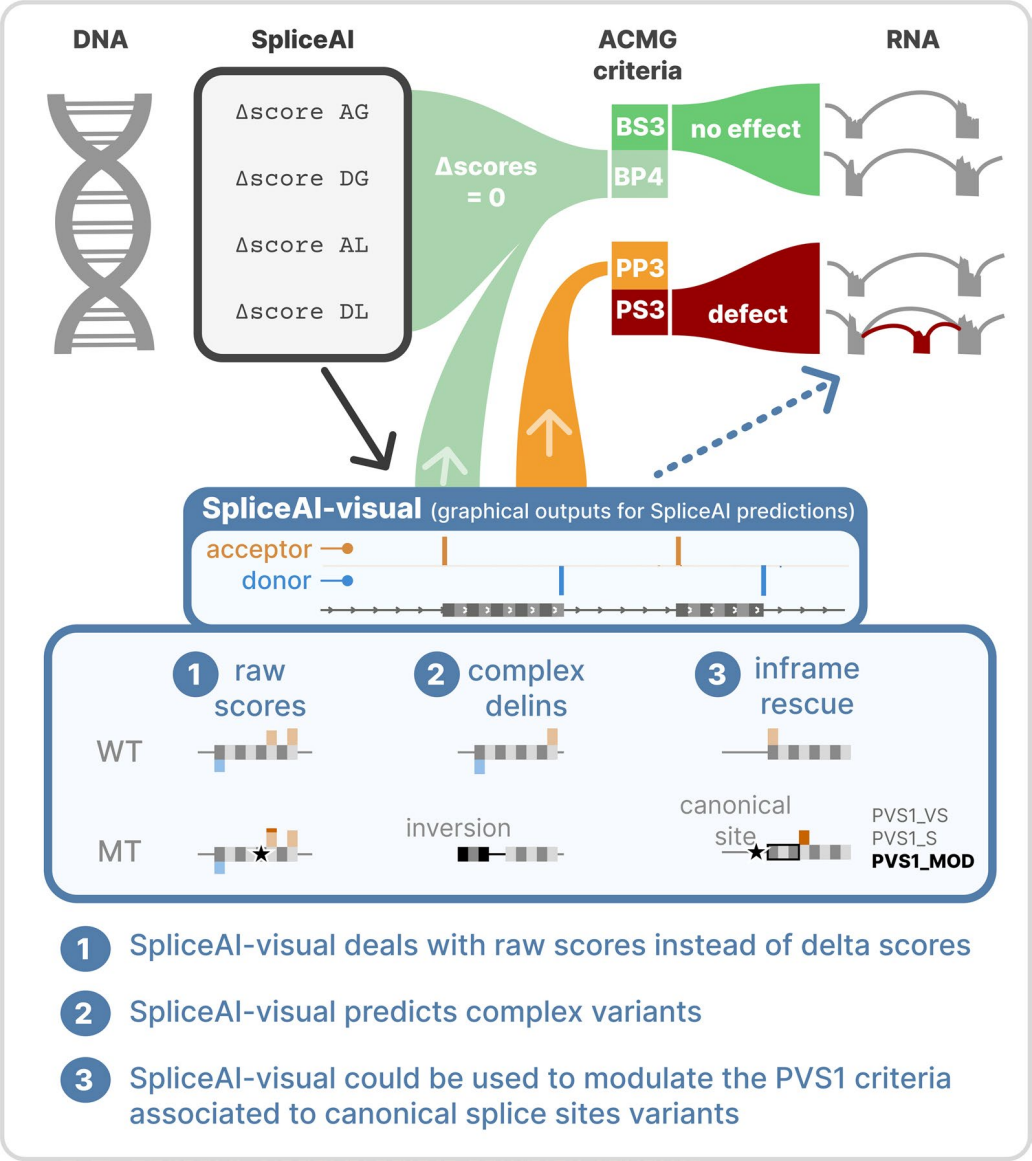

**Supplementary Information:**

The online version contains supplementary material available at 10.1186/s40246-023-00451-1.

## Introduction

Exome and genome sequencing currently identify on a daily basis many novel or uncharacterized variants worldwide. A significant proportion (up to 60% [1]) of the pathogenic variants identified are likely to alter the correct splicing of the transcript. However, the functional validation of a variant predicted to alter splicing requires in vitro tests or additional and sometimes invasive biological samples. These validations are often time-consuming and expensive. Therefore, there is a strong need for in silico tools that can facilitate the precise interpretation of candidate variants to (1) correctly prioritize the best candidates to be investigated, and (2) choose the optimal functional validation test according to the expected alteration. The efficiency of SpliceAI to predict a variant’s splicing alteration has been attested by multiple studies [2–10]. Furthermore, thanks to its neural network, SpliceAI is able to make predictions about the global splicing outcome (e.g., exon skipping, splicing rescue by cryptic site activation, pseudo-exon creation, etc.). This ability to focus not only on the nearby site (destruction or creation) but at the whole transcript level is a unique feature of these deep-learning-based next-generation splicing predictors, such as SpliceAI or Pangolin [11]. In a recent improvement, the SpliceAI neural network has been retrained with a curated and manually validated isoforms dataset [12]. Still, the standard version of SpliceAI (currently v1.3.1) has some limitations. First, predictions and relative positions of the altered splice sites are displayed as numerical values, which can be confusing when estimating which exact sites are altered, or when dealing with long-distance effects. Second, the results are the delta scores (DS) between the raw scores (RS) of the reference allele and the variant allele, which can be difficult to interpret and in some cases misleading, in particular when the reference value is comprised within the intermediate range of interpretation (i.e., [0.2–0.8]). Indeed, the DS provided by the genuine SpliceAI account for the maximal differences between the predictions of the variant and the reference allele, for the 4 predicted categories being acceptor gain (AG), acceptor loss (AL), donor gain (DG), and donor loss (DL). In the original publication describing SpliceAI, the DS cutoff of 0.2 has been characterized as a “permissive” threshold to retain splice-altering variants with high sensitivity [2]. Therefore, this threshold is widely used, but may filter out pathogenic variants if the difference is subtle (i.e., increase in an already high donor or acceptor site). Finally, SpliceAI current public implementations (e.g., spliceailookup, https://spliceailookup.broadinstitute.org/) or pre-computed whole genome VCFs only annotate simple variants (i.e., substitutions, insertions, deletions), prohibiting the interpretation of more complex deletions–insertions or inversions, with the notable exception of the recent CI-SpliceAI [12].

To overcome these limitations, we developed SpliceAI-visual, a simple and free-to-use online tool, based on the original SpliceAI model, which provides the SpliceAI’s RS. Available via a Google Colab notebook (https://tinyurl.com/spliceai-visual), the SpliceAI-visual predictions are graphically displayed on a dynamic window, and bedGraph files are downloadable for further analyses in a standard genome browser (compatible with IGV and UCSC Genome Browser) [13, 14]. In addition, the SpliceAI-visual solution has been implemented in MobiDetails (https://mobidetails.iurc.montp.inserm.fr/MD), a free online user-friendly DNA variant interpretation tool, and is displayed by default for any annotated variant [15]. Here, we validated the advantage of using SpliceAI-visual on variants from the literature and we show how it helped to identify new splicing-altering variants, to reconsider the loss-of-function prediction (i.e., modulating PVS1), and to interpret complex variants.

## Methods

In this study, we refer to "raw" scores (RS) for the absolute prediction of SpliceAI, in opposition to the "delta" scores (DS). We wish to dismiss any confusion concerning the "raw" scores found in the SpliceAI terminology, referring there to "raw" delta scores, in opposition to "masked" delta scores (see https://github.com/Illumina/SpliceAI for more details).

### SpliceAI-visual

For SpliceAI-visual, the SpliceAI model (https://github.com/Illumina/SpliceAI, custom sequence function) is run independently on two sequences (reference allele; variant allele), generating for each nucleotide its likelihood (RS) to be used as an acceptor or a donor site in a biological context. Results are then used to generate 2 bedGraph files (http://genome.ucsc.edu/goldenPath/help/bedgraph.html). In the Colab notebook, scores are computed for both the entire reference and variant transcripts in real time. The reference and variant bedGraph files can be loaded in a genome browser.

To integrate SpliceAI-visual in MobiDetails, we have used SpliceAI v1.3.1 to pre-compute the RS for 57,271 transcripts including 19,120 Matched Annotation by NCBI and EMBL-EBI (MANE) transcripts available on the Web site [16], using Illumina® models available for non-commercial usage (see https://github.com/Illumina/SpliceAI for more details). Then, RS predictions for the wild-type sequences for these full transcripts are stored as bedGraph files and are directly available for comparison with the variant RS predictions. RS predictions for the variant have to be computed in real time (the software architecture is described in Additional file 1: Fig. S1). The variant allele consists of 10 kb of genic sequence surrounding the variation (truncated if the variation is located less than 5 kb from the 3’ or 5’ end of the transcript). Indeed, the authors of SpliceAI have shown that their algorithm was the most accurate using 5 kb of DNA sequence surrounding the variant position [2]. We added an additional 5 kb on each side of the variant to display a larger picture of the splicing pattern of the region.

**Fig. 1:**
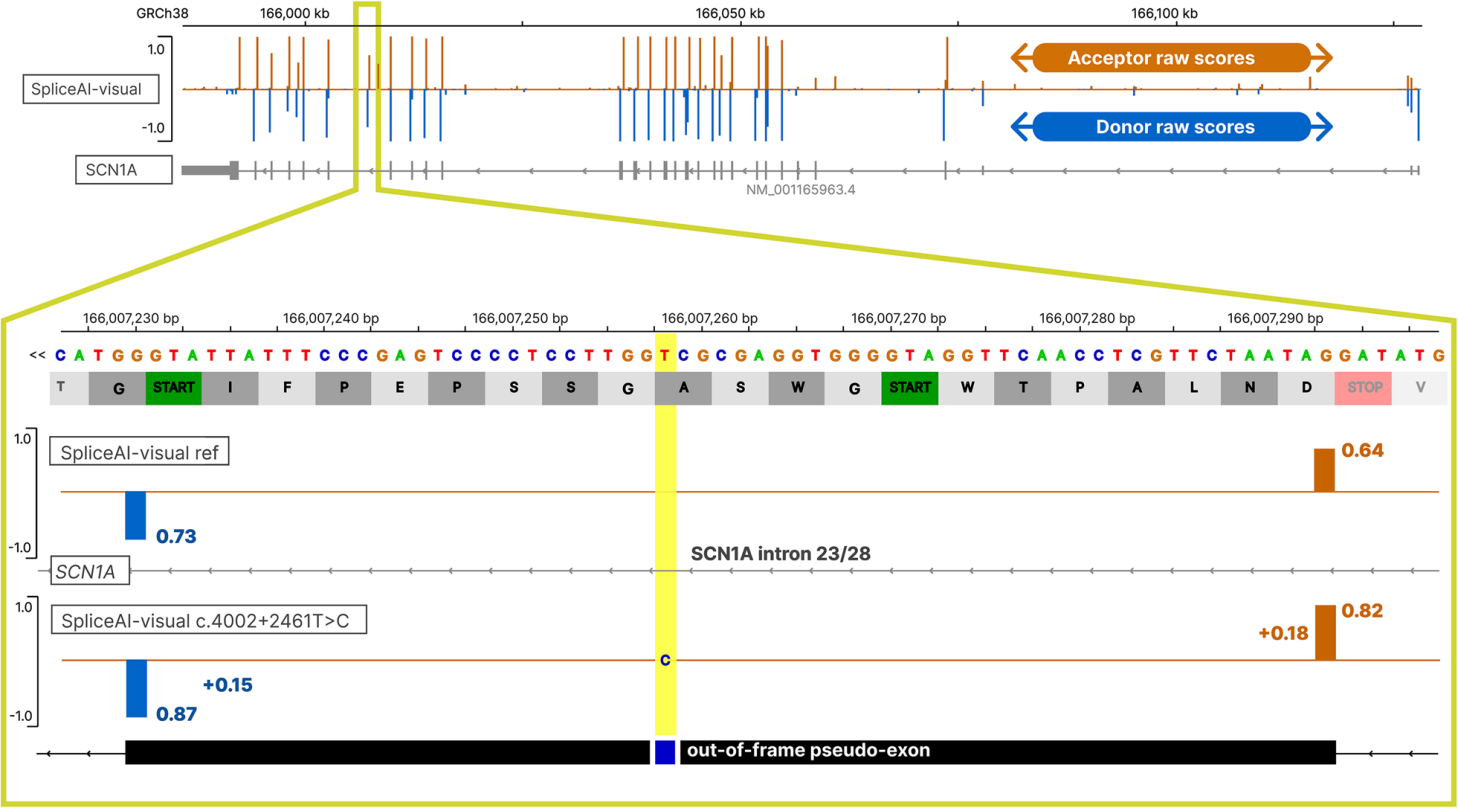
The delta score (DS) pitfall: discrepancy between SpliceAI’s DS and SpliceAI raw scores (RS). SpliceAI-visual outputs of *SCN1A* deep intronic variant displayed in IGV. Above: SpliceAI raw scores for the reference allele of *SCN1A*; below: SpliceAI RS for the pathogenic deep intronic variant NM_001165963.4(SCN1A):c.4002 + 2461 T > C functionally attested to cause the retention of an intronic retention of 64pb[REF]. Orange: acceptor site prediction; Blue: donor site prediction. The variant position is highlighted in yellow

A dedicated Flask API (https://palletsprojects.com/p/flask/) available in a private server (source code available at https://github.com/mobidic/spliceai) is asynchronously called by the public MobiDetails server to compute the variant allele RS (in about 30 s) (see Additional file 1: Fig. S1). Computation requests on the private server are handled by the Apache Web server (https://apache.org/) and queued with the SLURM workload manager (https://slurm.schedmd.com/). SpliceAI is run in CPU-only mode. The API returns JSON objects including the DNA sequence and the associated SpliceAI RS, which are converted into BedGraphs by the MobiDetails public server. BedGraphs are then displayed on the Web page within an igv.js genome browser (https://github.com/igvteam/igv.js/) as two separate tracks (reference and variant BedGraphs). A third track is optionally provided corresponding to the RS of the extra inserted nucleotides when the variant allele is longer than the reference allele. As an option, users can request in a simple click the prediction of the whole variant transcript, which is displayed in a dedicated track in the genome browser. In this case, the computation time directly depends on the size of the transcript (from seconds to several minutes).

### SpliceAI delta scores

The SpliceAI DS of the variants explored in this study were generated using SpliceAI v1.3.1, with the maximal window of ± 4999 bp surrounding the variant on the MANE select transcript.

### DNA, RNA, and plasma progranulin analysis

DNA sequencing and RNA sequencing were performed through various methods and protocols, as described in the Additional file 1: Methods. Briefly, DNA sequencing of the *SETD5* cases (patient 1 and 5) was performed by trio-based genome sequencing, DNA sequencing of patient 2 was performed by Sanger sequencing of the exons of *GRN*, DNA sequencing of patient 3 was performed by trio-based exome sequencing, DNA sequencing of patient 4 was performed by targeted gene sequencing (gene panel) and plasma progranulin levels were measured by ELISA, as described in the Additional file 1: Methods.

## Results

We developed SpliceAI-visual, which displays SpliceAI’s RS on a genome browser. SpliceAI-visual betterments compared to SpliceAI are summarized in Table 1.

**Table 1:** SpliceAI-visual solves some SpliceAI limitations

SpliceAI limitation	SpliceAI-visual enhancement
Numerical values	Graphical outputs, allowing fast and precise interpretation especially for PVS1 modulation
Delta scores pitfall	Raw scores
Complex delins not annotated	Complex delins annotated

### Overcoming the DS pitfall

As already stated, the value of 0.2 is recommended by the authors of SpliceAI as a threshold for the four DS to discriminate potential splice-altering variants from non-altering variants. We present several examples demonstrating the relevance of SpliceAI-visual when the DS are low.

### Examples from the literature

#### Identifying pseudo-exon inclusion

##### *SCN1A*

The deep intronic substitution NM_001165963.4(*SCN1A*):c.4002 + 2461 T > C (Table 2, Fig. 1) has been demonstrated by minigene assays to induce the exonization of an out-of-frame 64-bp intronic sequence [17]. This 64-bp exonization mechanism has not been elucidated, but was correctly identified by SpliceAI with low DS (AG: 0.18; DG: 0.15). Using SpliceAI-visual, we show that while the DS are below the recommended threshold, the RS for the wild-type sequence are already significant (acceptor site: 0.64; donor site: 0.73). This results in high RS for the variant sequence T > C (acceptor site: 0.82; donor site: 0.87) and finally in the inclusion of the intronic sequence in the transcript. The mRNA proportion aberrant/normal transcript was not estimated.

**Table 2:** HGVS descriptions, SpliceAI, SpliceAI-visual scores, and ACMG classification of the variants analyzed in this study

Gene symbol	Coding/protein	SpliceAI DS(DP)*	SpliceAI-visual RS(DP)**	Category/Reference	ACMG
*SCN1A*	NM_001165963.4:c.4002 + 2461 T > C p.?	AG = 0.18 (35) AL = 0 DG = 0.15 (− 28) DL = 0	Ref: A = 0.64 (35), D = 0.73 (− 28) Var: A = 0.82 (35), D = 0.87 (− 28)	DS pitfall pseudo-exonization/Li et al., 2021	Likely pathogenic (PS3, PM2, PP1)
*MFGE8*	NM_005928.4:c.871-803A > G p.?	AG = 0.15 (144) AL = 0 DG = 0.16 (43) DL = 0	Ref: A = 0.68 (144), D = 0.58 (43) Var: A = 0.84 (144), D = 0.74 (43)	DS pitfall Yamaguchi et al., 2010	Not Applicable (candidate gene)
*SETD5*	NM_001080517.3:c.2476 + 198A > C p. ?	AG = 0.05 (− 61) AL = 0 DG = 0.04 (35) DL = 0	Ref: A = 0.94 (− 61), D = 0.96 (35) Var: A = 0.99 (− 61), D = 0.99 (35)	DS pitfall This study	Pathogenic (PS3, PS2, PM2)
*GRN*	NM_002087.4:c.-9A > G p.?	AG = 0 AL = 0 DG = 0.19 (272) DL = 0.48 (1)	Ref: D = 0.94 (1) Var: D = 0.44 (1)	DS pitfall This study	Likely pathogenic (PS3, PP5, PM2, PP4)
*CASK*	NM_001367721.1:c.172 + 1G > A p. ?	AG = 0 AL = 0 DG = 0.71 (− 17) DL = 0.99 (1)	Ref: D = 0 (1), D = 0.29 (− 17) Var: D = 0 (1), D = 0.99 (− 17)	Adjusting PVS1 Intronic retention of 18 bp leading to the in-frame insertion of 6 amino acids r.172_173ins[172 + 1_172 + 18] p.(Asp58delinsGlyLysArgTrpIleSerAsn) This study	Uncertain significance (PVS1_M, PM2)
*KMT2D*	NM_003482.4:c.5189-1G > C p.?	AL = 0.98 (− 1) DL = 0 AG = 0.95 (− 25) DG = 0.07 (− 253)	Ref: A = 1 (1), A = 0.04 (− 25) Var: A = 0.02 (1), A = 0.99 (− 25)	Adjusting PVS1 11 individuals in UK Biobank exomes, prediction of the in-frame deletion of 8 amino acids	Uncertain significance (PVS1_M, PP5, BS2)
	NM_003482.4:c.5782 + 1G > A p.?	AL = 0 DL = 1 (1) AG = 0.01 (− 431) DG = 0.28 (− 8)	Ref: D = 1 (1), D = 0.71 (− 8) Var: D = 0 (1), D = 1 (− 8)	Adjusting PVS1 3 heterozygous individuals in gnomAD, prediction of the in-frame insertion of 3 amino acids	Uncertain significance (PVS1_M, PP5, BS2)
*TTN*	NM_001267550.2:c.31349-1G > C p. ?	AG = 0.51 (− 10) AL = 0.97 (− 1) DG = 0 (43) DL = 0 (1)	Ref: A = 0.97 (− 1), A = 0.02 (− 10), D = 0.99 (− 78) Var: A = 0 (− 1), A = 0,53 (− 10), D = 0.80 (− 78)	Adjusting PVS1 In-frame rescue acceptor site, leading to the loss of 9 bp This study	Uncertain significance (PVS1_M, PM2)
*SETD5*	NM_001080517.3:c.568-31_568dup p.(Asn190Ilefs*20)	AG = 0.07 (0) AL = 0.01 (− 3574) DG = 0 DL = 0.01 (135)	Ref: A = 0.99 (31), D = 0.98 (278) Var: A = 0.99 (31), D = 0.98 (278)	Adjusting PVS1 This study	Benign (PM2, BS3, BS4) *Of note, PVS1 has been excluded*
*EYS*	NM_001142800.2:c.2992_2992 + 6delinsTG p.?	Not supported	Ref: A = 0.97 (151), D = 0.99 (6) Var: A = 0.13 (151), D = 0 (6)	Complex delins Westin et al. 2021	Likely pathogenic (PS3, PM2, PM3)

*SpliceAI: delta score (DS), delta position (DP), acceptor gain (AG), acceptor loss (AL), donor gain (DG), donor loss (DL)

**SpliceAI-visual: Raw scores (RS), delta position (DP) for acceptor A(DP) or donor D(DP) sites, for reference (Ref) and variant (Var) alleles

##### *MFGE8*

**Fig. 2:**
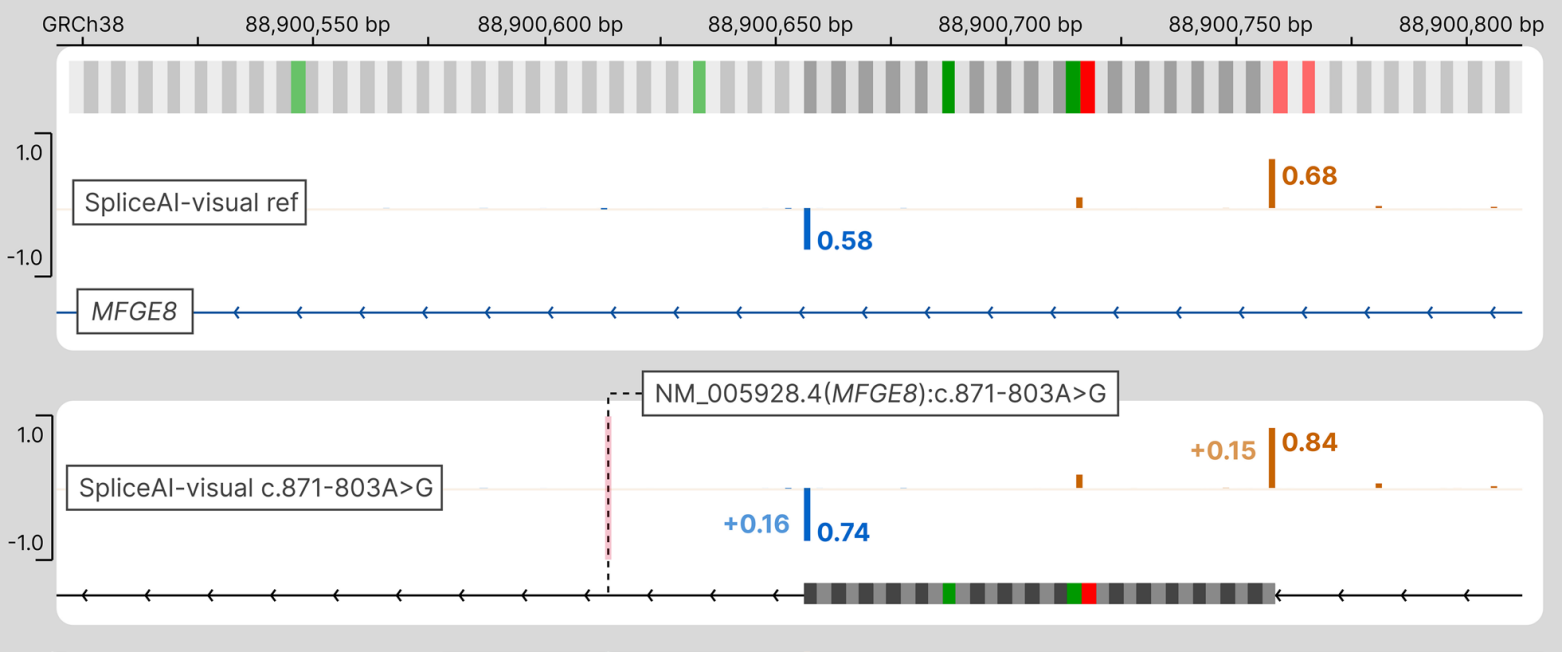
The delta score (DS) pitfall: discrepancy between SpliceAI’s DS and SpliceAI raw scores (RS). SpliceAI-visual outputs of *MFGE8* deep intronic variant displayed in IGV. Above: SpliceAI RS for the reference allele of *MFGE8*; below: SpliceAI RS for the pathogenic variant NM_005928.4(*MFGE8*):c.871-803A > G functionally attested to cause the exonization of an intronic sequence containing a stop codon (red). Orange: acceptor site prediction; Blue: donor site prediction. The variant position is pointed by a dashed line

Similarly, the pathogenic variant NM_005928.4(*MFGE8*):c.871-803A > G is responsible for the inclusion of an intronic sequence containing a stop codon (Table 2, Fig. 2) [18]. Again, the SpliceAI DS are low (AG: 0.15; DG: 0.16), but the reference allele was already identified with mild RS.

SpliceAI-visual identified the resulting acceptor and donor sites on the variant allele as strong candidates (respectively, 0.84 and 0.74), and the use of a graphical output (bedGraph files) loaded in a genome browser allowed a quick identification of the termination codon using the three frames translation track in IGV or in the UCSC Genome Browser. This intronic inclusion was estimated to be ~ 10 times more abundant than the wild-type transcript.

#### Unpublished cases

##### *SETD5*: enhancing the retention of a “poison” exon

**Fig. 3:**
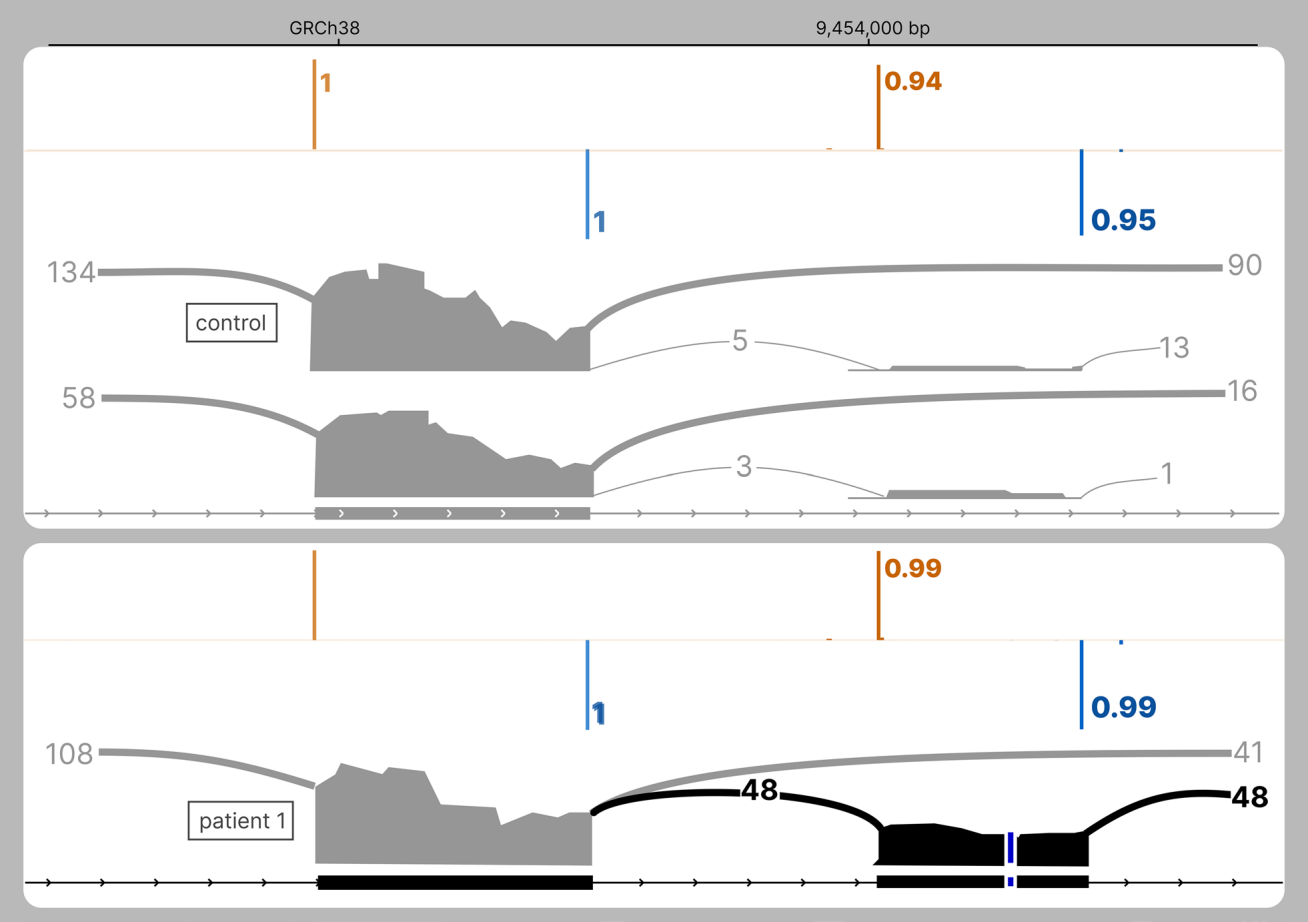
The delta score pitfall: *SETD5* poison exon retention caused by an intronic substitution. RNAseq and SpliceAI-visual outputs displayed in IGV. Above: SpliceAI RS for the reference allele of *SETD5*, along with one control individual; below: SpliceAI RS for the pathogenic deep intronic variant NM_001080517.3:c.2476 + 198A > C, along with RNAseq of patient 1. Orange: acceptor site prediction; Blue: donor site prediction. The variant position is pointed out with a dashed line. Although the variant A > C is heterozygous, 95% of RNAseq reads carry the C, suggesting the causative role of this allele in the retention

Genome-trio sequencing of patient 1 revealed a de novo variant in intron 17 of *SETD5*: NM_001080517.3:c.2476 + 198A > C (Table 2, Fig. 3). SpliceAI DS were low with an AG and DG of 0.05 and 0.04, respectively. However, those DS were added to high RS (acceptor: 0.94, donor: 0.95) as shown by SpliceAI-visual. Indeed, we observed a low level of intronic retention in RNAseq of controls. This intronic retention of 97 bp led to the inclusion of a premature stop codon and a presumed degradation by NMD. By performing RNAseq from a blood sample of the patient, we showed that the intronic retention of this “poison” exon was dramatically enhanced compared to 2 controls. The variant was found in 95% of the reads, confirming the causal effect of our variant on this retention.

##### *GRN*: guiding functional investigations

**Fig. 4:**
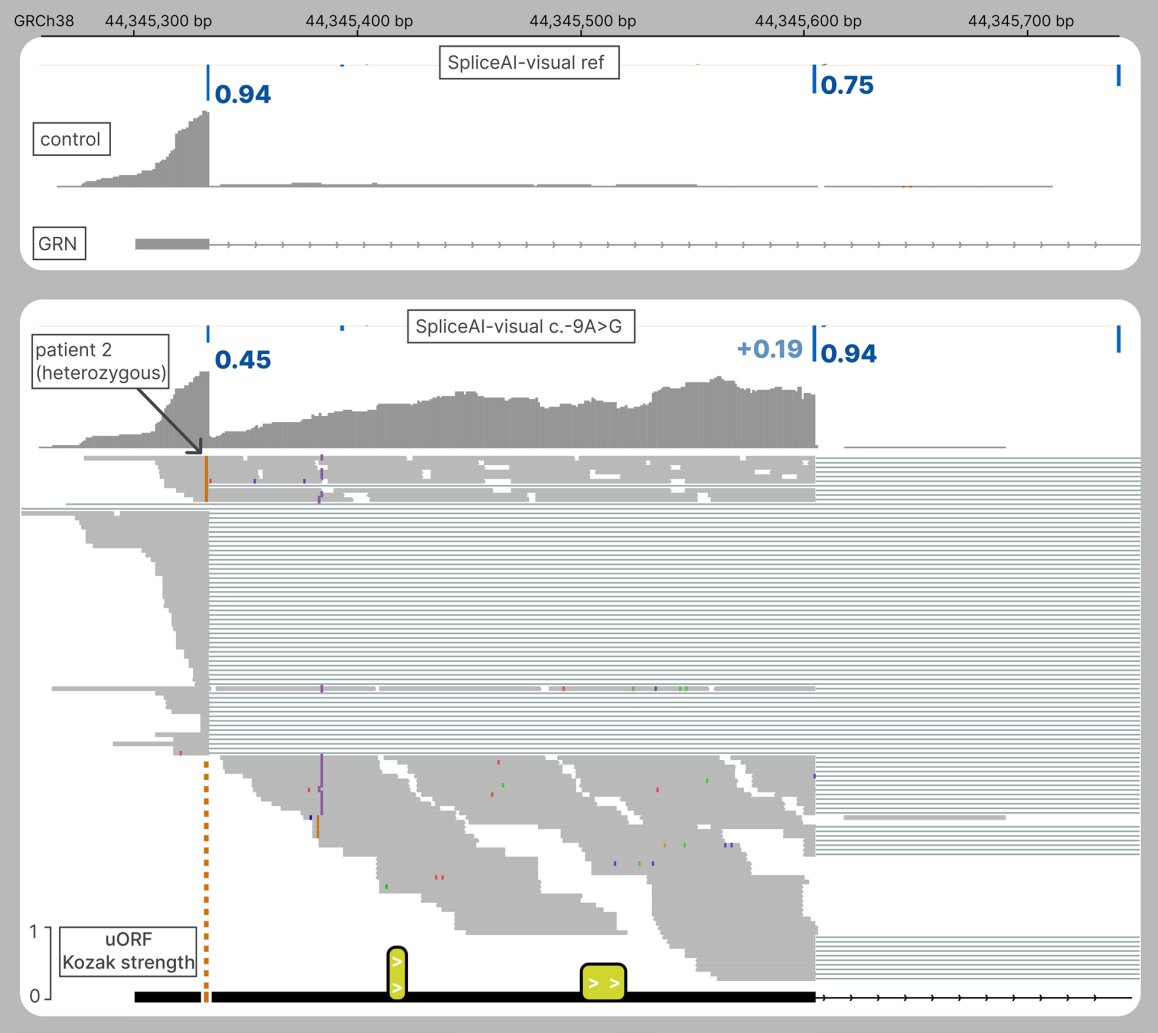
The delta score pitfall: extending the 5’UTR of *GRN*. RNAseq and SpliceAI-visual outputs displayed in IGV. Above: SpliceAI RS for the reference allele of *GRN* along with RNAseq from one control; below: SpliceAI RS for NM_002087.4(*GRN*):c.-9A > G, along with RNAseq of patient 2. Bottom: two upstream Open Reading Frames in the intronic retention (yellow), height corresponding to the initiation strength of the AUG codon based on the Kozak context from TIS [20]

SpliceAI-visual is also convenient for guiding functional investigations. The following heterozygous variant NM_002087.4(*GRN*):c.-9A > G (Table 2) was identified in a 70-year-old male with Fronto-Temporal Dementia (patient 2), and plasmatic progranulin values compatible with a monoallelic alteration of *GRN* (see Sup Methods and Patients). This variant was previously identified in another affected patient, but the authors failed to evidence any abnormal splicing products [19]. This variant is predicted by SpliceAI to weaken the canonical donor site of this first 5’UTR exon (donor loss of 0.48). The initial RT-PCR has been performed on fibroblasts, but the exonic primers (F1-R1) failed to identify any abnormal products, as previously reported, even in the presence of an NMD inhibitor. Thanks to SpliceAI-visual, we were able to spot the putative rescuing donor site, which was predicted with a modest gain of + 0.19, but added to an RS of 0.75 on the reference allele (Fig. 4). This prediction was in favor of a 271-bp intronic retention. Another reverse primer (R2) has been designed in the predicted intronic 271-bp retention and showed amplification in the patient, and not in control individuals. The failure of the initial exonic RT-PCR (F1-R1) to amplify both wild-type and retention fragments could be due to the competitive advantage of the short fragment over the fragment including the 271-bp retention.

#### Adjusting the PVS1 criteria

According to the standard guidelines of the American College of Medical Genetics and Genomics (ACMG), the PVS1 criteria includes “canonical +/− 1 or 2 splice sites in a gene where the loss of function is a known mechanism of disease” [21]. However, alteration of a canonical splice site can result in other non-truncating consequences by various mechanisms: (1) an in-frame exon skipping (initially stated in the caveats of the aforementioned guideline), (2) an in-frame deletion by the creation of an exonic rescuing splice site, or (3) an in-frame intronic retention devoid of in-frame stop codon [22, 23]. We show here with various cases the relevance of SpliceAI-visual in the assessment of the PVS1 criteria relative to variants altering canonical splice sites.

##### *CASK*

**Fig. 5:**
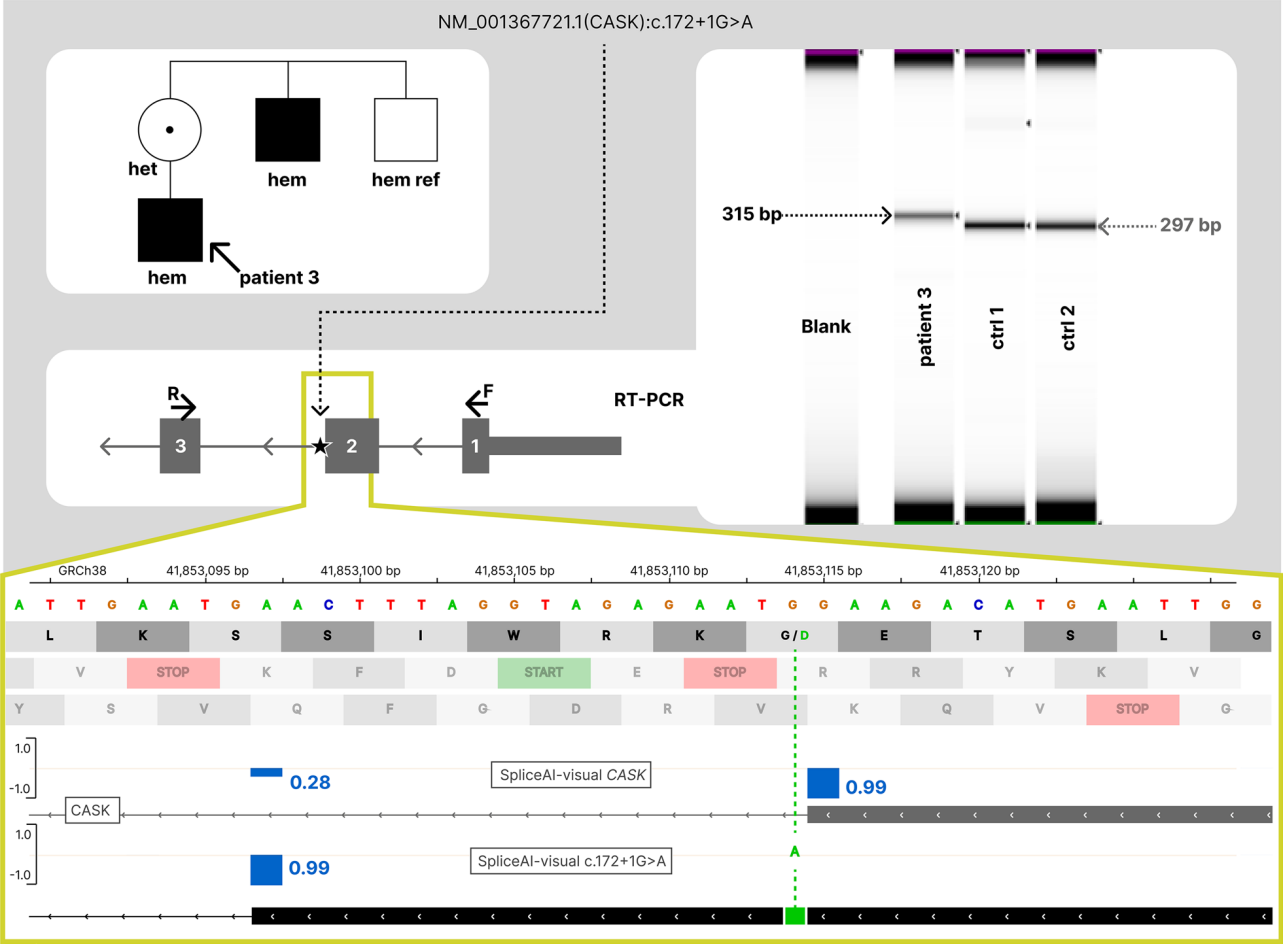
Scaling down the PVS1 criteria of a canonical splice site variant in *CASK*. Segregation, RT-PCR and SpliceAI RS of NM_003688.3(*CASK*):c.172 + 1G > A, hemizygous in patient 3. This variant leads to the complete in-frame retention of 18 bp (no wild-type 297 bp product was observed in patient 3 RT-PCR lane), as predicted by SpliceAI-visual. This 18-bp retention does not include stop codon and is predicted to insert 6 amino acids

We report the case of a 9-year-old male individual, presenting with learning disabilities and microcephaly (see Additional file 1: Methods and Patients, patient 3). Solo-exome sequencing showed a hemizygous substitution in a canonical donor site of the gene *CASK*, NM_003688.3(*CASK*):c.172 + 1G > A, absent from control databases (gnomAD, deCAF) [24, 25]. No other pathogenic or likely pathogenic variant was retained. This donor site disruption affects the MANE transcript of *CASK*. This hemizygous variant of patient 3 is predicted by SpliceAI to result in a DL, along with a + 0.71 DG. With SpliceAI-visual, this DG was predicted to lead to in-frame retention of 18 bp (6 amino acids, no stop codon, Fig. 5). Furthermore, this donor’s DS of + 0.71 adds to a probability of 0.28 on the reference allele, resulting in an RS of 0.99 on this donor site (Fig. 5). In accordance with SpliceAI-visual predictions, RT-PCR on peripheral blood of patient 3 identified the 18-bp retention on 100% of transcripts (Fig. 5), which precluded the use of the Very_Strong weight of the PVS1 criteria. Without the very strong weight, this variant couldn’t be classified as likely pathogenic or pathogenic. The significance of this variant was classified as Uncertain (Table 2).

##### KMT2D

**Fig. 6:**
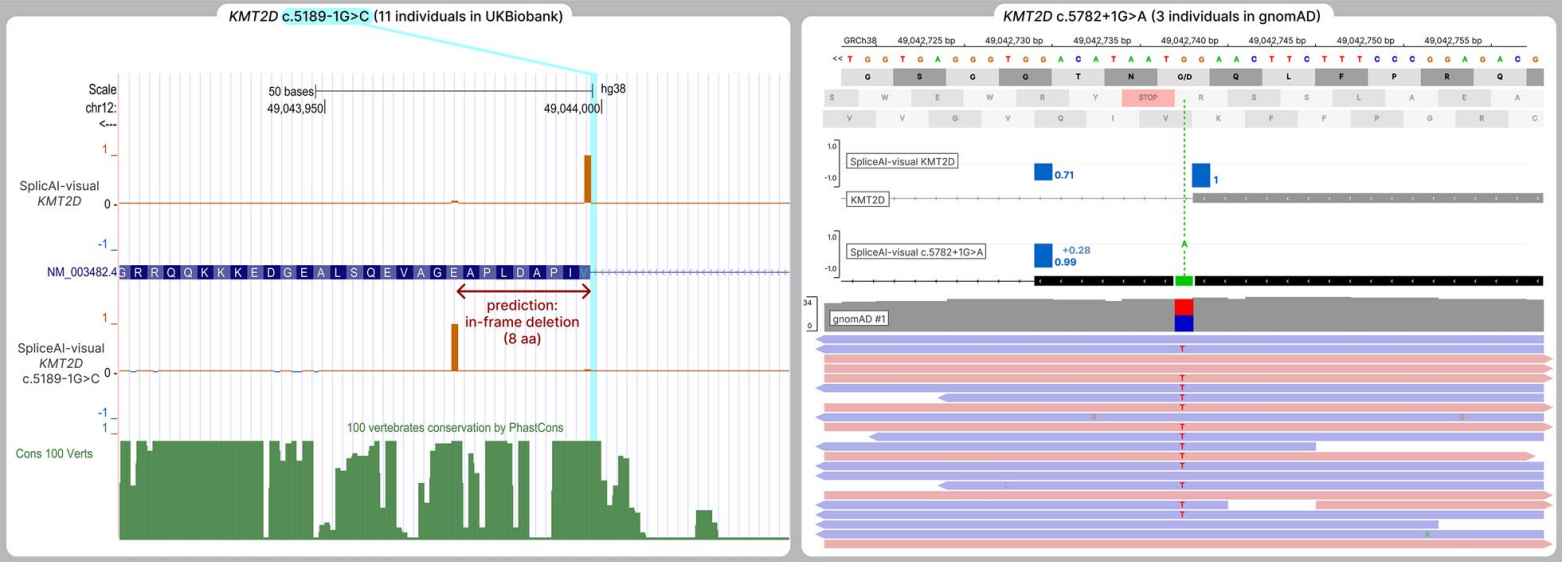
Scaling down the PVS1 criteria of canonical splice site variants in *KMT2D*. Left: another a priori PVS1 variant NM_003482.4(*KMT2D*):c.5189-1G > C, present in 11 individuals in UK Biobank. This variant is predicted to result in an in-frame rescuing acceptor site, deleting 8 poorly conserved amino acids. Right: SpliceAI-visual outputs and BAM from one heterozygous from gnomAD of NM_003482.4(*KMT2D*):c.5782 + 1G > A. This variant is present in 3 individuals in gnomAD, which is not consistent with the penetrance of loss-of-function variants of *KMT2D*. Also, the mild rescuing DS of 0.28 is added to a nonzero RS on the reference allele (delta score pitfall) and is predicted to result in a complete rescue of this donor site, with the in-frame retention of 9 bp

The variants NM_003482.4(*KMT2D*):c.5189-1G > C and c.5782 + 1G > A (Table 2) are located in canonical splice sites of *KMT2D* and solely on this argument, the PVS1 criteria could apply, as loss-of-function variants are a known mechanism of *KMT2D*-related Kabuki syndrome. Based on this argument, these variants have recently been submitted as Likely Pathogenic in ClinVar (VCV001496460.1, VCV001506261.1) [26]. Surprisingly, these variants were reported in unaffected individuals in the general population (c.5189-1G > C is absent from gnomAD v2.1.1 / v3.1.2, but found in 11 individuals in UK Biobank exomes [24, 27]. c.5782 + 1G > A is present in 3 heterozygous individuals in gnomAD v2 and v3) [24], which is inconsistent with the penetrance and severity of monoallelic *KMT2D* loss-of-function variants (OMIM: 147,920). This discrepancy could be explained by splicing rescue, which was well predicted by SpliceAI-visual (Fig. 6).

- For c.5189-1G > C, SpliceAI-visual shows the creation of an in-frame rescuing acceptor site, predicted to delete 8 poorly conserved residues.
- For c.5782 + 1G > A, SpliceAI-visual predicts the complete loss of the donor site (− 1), and a modest gain of an in-frame nearby donor site (+ 0.28). This modest gain is another example of the DS pitfall (see above), adding on to a cryptic site predicted with an RS of 0.71 on the reference allele, resulting in an RS of 0.99 on the alternate allele. Moreover, this donor-rescuing site results theoretically in the inclusion of 3 amino acids in the final product, which may have less deleterious consequences and explain the presence of this variant in gnomAD.

For c.5189-1G > C, SpliceAI-visual shows the creation of an in-frame rescuing acceptor site, predicted to delete 8 poorly conserved residues.

For c.5782 + 1G > A, SpliceAI-visual predicts the complete loss of the donor site (− 1), and a modest gain of an in-frame nearby donor site (+ 0.28). This modest gain is another example of the DS pitfall (see above), adding on to a cryptic site predicted with an RS of 0.71 on the reference allele, resulting in an RS of 0.99 on the alternate allele. Moreover, this donor-rescuing site results theoretically in the inclusion of 3 amino acids in the final product, which may have less deleterious consequences and explain the presence of this variant in gnomAD.

##### *TTN*

**Fig. 7:**
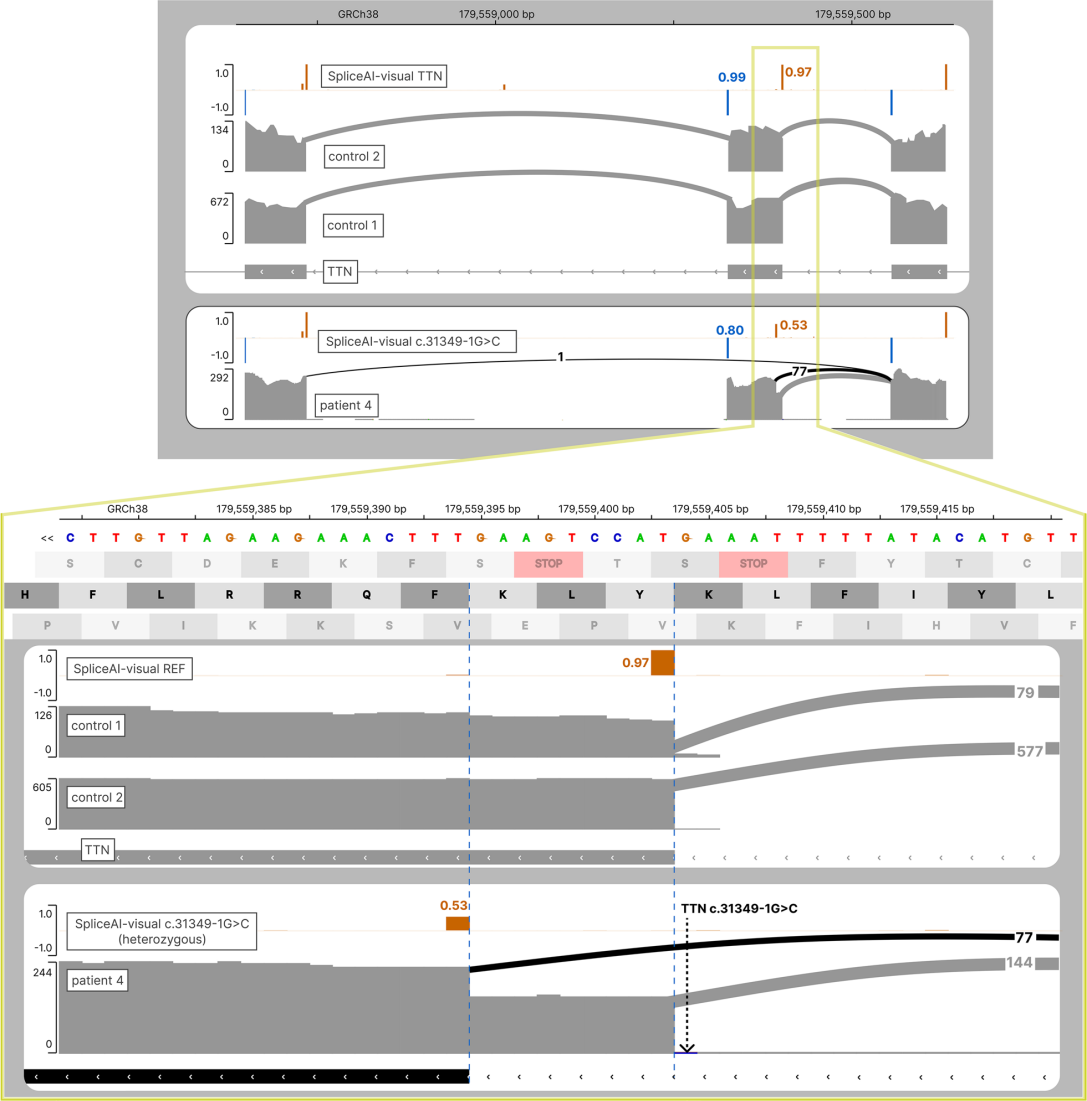
Scaling down the PVS1 criteria of a canonical splice site variant in *TTN*. RNAseq and SpliceAI-visual outputs displayed in IGV showing the predicted exon skipping (top view), and the in-frame rescue (bottom view). Top tracks: SpliceAI RS for the reference allele of *TTN* along with RNAseq from 2 controls; bottom tracks: SpliceAI RS for the NM_001267550.2(*TTN*):c.31349-1G > C along with RNAseq of patient 4

We describe here a similar case occurring in the *TTN* gene. NGS analyses targeted on congenital myopathy and muscular dystrophy gene panels identified in patient 4 (see Suppl. Methods for the phenotypic description) a variant in intron 116 of *TTN*: NM_001267550: c.31439-1G > C (Table 2) absent in the general population (gnomAD, deCAF) [24, 25] and predicted to affect splicing in exon 117. This variant located in the exon/intron junction of exon 117 is predicted to completely abolish the natural acceptor site, whereas the graphical output of SpliceAI-visual clearly shows a cryptic acceptor site located 9-bp downstream of the natural site (Fig. 7). Its use would lead to a 9-bp in-frame loss in exon 117, which has been confirmed by the RNAseq experiments (77 reads supporting the cryptic junction out of 222 reads (34.6%). Interestingly, SpliceAI-visual reveals a non-total raw probability of 0.53 to this rescuing acceptor site. Moreover, SpliceAI predicts the reduced strength of the natural donor site, located on the other side of exon 117. Taken together, these elements suggest a partial skipping of exon 117, which is further supported experimentally, as the exon 116–118 junction is attested by one read on RNAseq, and not seen in the two controls (Fig. 7). In the absence of a parental segregation study (no parents available) for dominant hypothesis, and of a second identified variant for recessive hypothesis, and regarding the RNAseq results, this variant was classified as a variant of uncertain significance (class 3).

##### *SETD5*

**Fig. 8:**
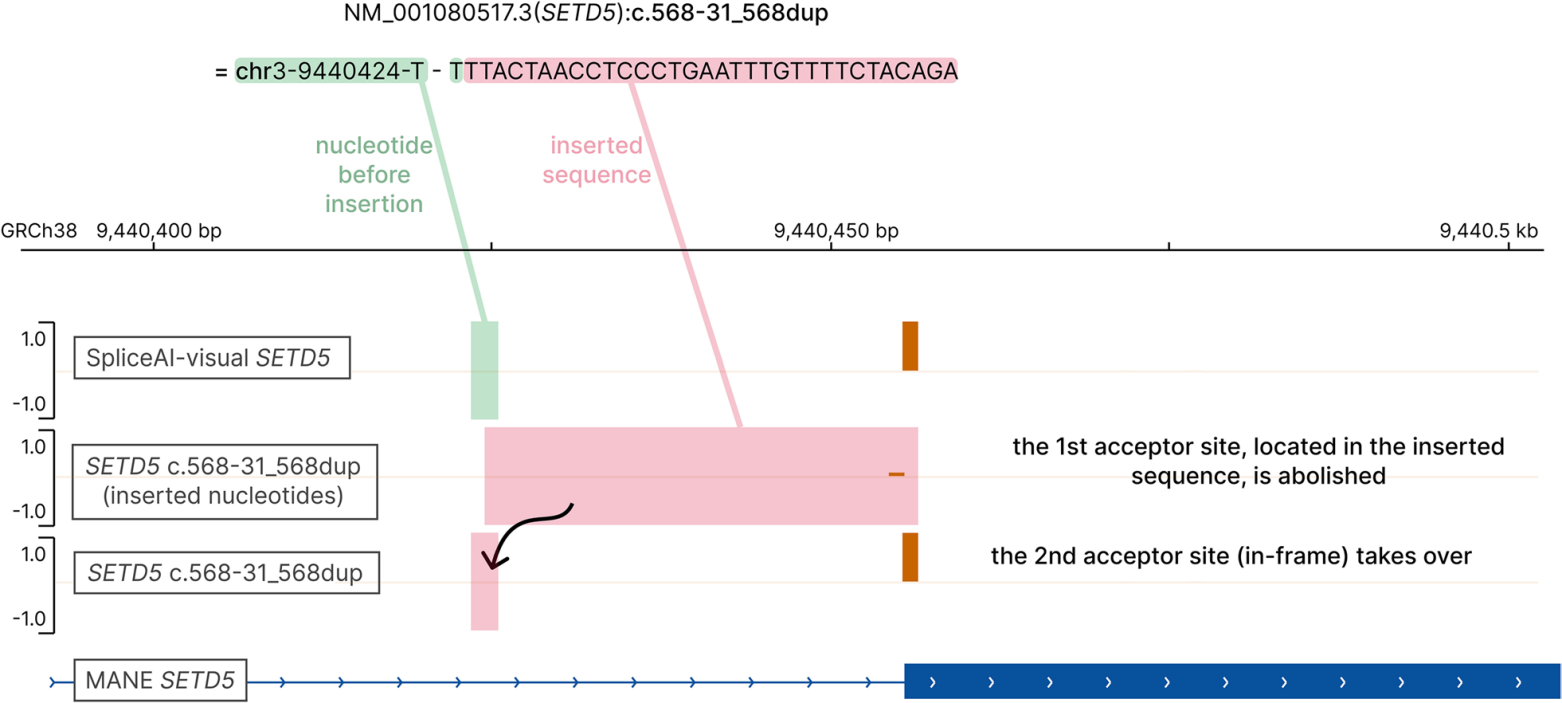
Scaling down the PVS1 criteria of a putative frameshift in *SETD5*. SpliceAI-visual outputs displayed in IGV showing the predicted benign splicing outcome of this putative frameshift

The following variant in *SETD5* was identified in patient 5 in the heterozygous state, NM_001080517.3(*SETD5*):c.568-31_568dup p.(Asn190IlefsTer20) (Table 2), inherited from his asymptomatic mother. This 31-bp duplication is absent from gnomAD or deCAF [24, 25]; it duplicates the exon–intron border of exon 8 of *SETD5* and is considered to have a high truncating impact according to SNPEff and VEP annotators [28, 29]. Indeed, this variant duplicates the acceptor site, resulting in two competing nearby acceptor sites: the first being out-of-frame—hence the predicted frameshift—and the second being in-frame. SpliceAI-visual, however, shows the second site to be the strongest, predicting no splicing alteration (Fig. 8), which was confirmed by RNAseq.

#### Interpreting complex delins

**Fig. 9:**
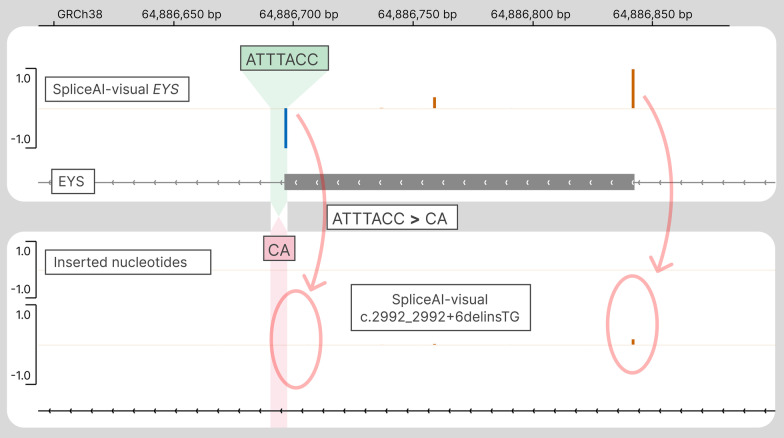
SpliceAI-visual outputs displayed in IGV showing the predicted exon skipping resulting from the complex delins NM_001142800.2(*EYS*):c.2992_2992 + 6delinsTG. Top track: SpliceAI RS for the reference allele of *EYS*; bottom track: SpliceAI RS for the delins in *EYS*

Finally, SpliceAI-visual allows the interpretation of complex variants. For example, the following variant is a complex deletion–insertion variant occurring on an exon–intron border in the gene NM_001142800.2(*EYS*):c.2992_2992 + 6delinsTG (Table 2). However, most SpliceAI current public implementations or pre-computed whole genome VCFs currently do not process complex delins variations (i.e., other than deletion, insertion, or substitution), nor does Pangolin. Of note, those complex variations are handled by CI-SpliceAI but with numerical results [12]. The functional study of this variant by a minigene assay has shown the skipping of an entire out-of-frame exon [30]. We show that this exon skipping is well predicted by SpliceAI-visual (Fig. 9). In addition, we have tested SpliceAI-visual’s ability to predict 13 other complex delins, all of which were functionally attested to alter splicing, and correctly predicted by SpliceAI-visual (Additional file 1: Table S1).

## Discussion

Functional validation of putative splice-altering variants is often difficult and resource-consuming. Also, besides their accessibility, specific RT-PCR, RNA sequencing or minigene assays all have their limitations (e.g., primer design, tissue expression, restricted to middle exon, etc.4). Given the growing number of putative splice-altering variants identified by large genome sequencing, the decision to perform such functional splicing assays is not trivial. The relevance of prediction tools to filter and to accurately evaluate a variant’s expected splicing outcome is crucial.

We have shown that the DS of SpliceAI’s predictions could in certain cases be misleading, and have introduced the relevance of interpreting splicing predictions with RS, as a complementary analysis.

The threshold of 0.20 used for DS has been qualified as “relatively permissive” and as a “high recall” threshold by the original authors of SpliceAI (https://github.com/Illumina/SpliceAI).2 However, the three deep intronic pathogenic or likely pathogenic splicing variants of *SCN1A*, *MFGE8*, and *SETD5* would have been filtered out with this threshold. SpliceAI-visual represents a convenient manner to predict the splicing outcomes of these variants.

Interestingly, the authors of SpliceAI observed a decreased sensitivity of SpliceAI to predict the splice alterations of deep intronic variants, compared to variants located near exons. This was also recently reported for Pangolin [11]. They hypothesized this phenomenon to be caused by a putative intronic deprivation of specific markers, which are usually enriched near exons by selection. This diminished performance of SpliceAI in deep introns could also be partly explained by the pitfall of the DS approach. A recent study has shown a depletion of competitive decoy donors near the exon–intron junction [31]. If we hypothesize this donor site depletion to similarly affect acceptor sites, it is easy to think of introns as enriched of such dormant cryptic splice sites, as shown in Fig. 1. These cryptic intronic sites would be detected by SpliceAI, with non-null value in the reference allele, introducing an intronic bias for higher reference allele scores, and lower DS.

The need to access SpliceAI RS has been manifested in a recent study, aimed at predicting the activation of donor cryptic sites by a variant [31]. In line with this study, we believe that special caution should be taken into consideration when assessing the PVS1 criteria related to canonical position splicing outcomes. Indeed, splice alterations at these positions may lead to consequences differing significantly from a truncating variant, meaning typically in-frame insertion of a few nucleotides [22, 23, 32].

Concerning patient 3, according to the ACMG guidelines, the variant NM_003688.3(*CASK*):c.172 + 1G > A meets a priori the loss-of-function criteria (PVS1). However, patient 3 presented only a mild intellectual disability (see Patients and Methods), in striking contrast to the other patients reported with *CASK* loss-of-function variants. To our knowledge, only female patients have been reported with loss-of-function variants in *CASK*, all with severe developmental delay. Some male patients have been reported with truncating variants, but they were mosaic [33, 34]. Interestingly, four affected males were reported with a canonical acceptor site NM_001367721.1(*CASK*):c.2521-2A > T along with a mild phenotype. RT-PCR showed two in-frame deletions (an in-frame exon skipping—28 amino acids—and a 3 amino acid deletion), inconsistent with the loss-of-function criteria, PVS1 [35]. Of note, both of these in-frame deletions were predicted by SpliceAI-visual. We decided not to apply the PVS1 criteria for NM_003688.3(*CASK*):c.172 + 1G > A in patient 3, based on the RT-PCR amplification of the predicted 18-bp retention. In addition, NM_003688.3(*CASK*):c.172 + 1G > A was inherited from the asymptomatic mother, found at the hemizygous state in one symptomatic uncle with learning disabilities and absent from another asymptomatic uncle. This variant is currently classified as VUS, although it cannot be ruled out that this insertion of 6 amino acids is mildly deleterious at the hemizygous state, which would be consistent with the four affected males previously reported, along with the familial segregation analysis.

Using SpliceAI-visual when interpreting variants at canonical splice sites may avoid potential misinterpretation of their consequences, and allow correct prediction of the effect at the RNA level. Of course, the functional validation of the predicted effect remains necessary; however, if an in-frame consequence is clearly expected by SpliceAI and SpliceAI-visual, we propose to modulate the weight associated with the PVS1 criteria, following ClinGen Sequence Variant Interpretation Workgroup [36]^(p1)^. In addition to variants at canonical splice sites, the strength of the PVS1 criteria may also be modulated for predicted PTCs. Indeed, many putative PTCs have been reported to impact splicing, with in-frame consequences, associated with milder, or partial rescue of the associated phenotype [22, 23, 32, 37, 38].

Monoallelic alterations of the *SETD5* gene are implicated in intellectual disability, combining delayed psychomotor development and poor language development (OMIM #615761). The duplication of a natural splice site in *SETD5* identified in patient 5 in the heterozygous state, absent from the gnomAD database, and annotated as frameshift would have been consistent with the previous descriptions, where the intellectual disability is often mild. This variant was inherited from the asymptomatic mother, but this has been previously described for other pathogenic *SETD5* variants [39]. Thanks to SpliceAI-visual, the benign splicing outcome of this presumed frameshift duplication could be suspected and was further confirmed by RNAseq. The variant was then assumed to be probably benign.

SpliceAI-visual has also been useful to guide functional exploration in the *GRN* case, as it enabled the correct design of RT-PCR primers specific to the intronic retention. *GRN* RNAseq was consistent with monoallelic retention. Indeed, the exonic heterozygous c.-9A > G is only supported by reads aligned in the intronic retention, suggesting a total effect on splicing. Indeed, the low allele fraction observed on the sequence reads is presumably due to the 3’ bias of polyA mRNAseq, according to which the depth of the coverage decreases as the distance from the polyA tail increases. As the mRNA carrying the variant is shifted 271 bp after the intronic retention, it is more distant from the polyA than the wild-type mRNA at the position of the variant. As a consequence, this 271-bp difference in distance from the polyA results in a deeper coverage of the wild-type mRNA, relative to the mutated mRNA at the variant site. As to the mechanism by which this 271-bp intronic retention leads to a reduced amount of PGRN, we propose the following hypothesis. As previously described, the amount of transcript has been found to be similar in the presence or in the absence of nonsense-mediated decay (NMD) inhibitor, suggesting a limited NMD effect [19]. Interestingly, the retention included two AUG codons with moderate potential to initiate translation, as their Kozak consensus sequence strength was similar to that of the natural AUG of *GRN*. As small upstream open reading frames (uORF) can reduce the translation efficiency of a transcript, we hypothesize that these uORFs caused a nearly complete extinction of translation in the transcript including the retention [40].

SpliceAI-visual is also useful to assess the splicing outcomes of complex variants such as deletions/insertions, as, apart from running a private instance of SpliceAI, this is currently the only tool that computes such SpliceAI predictions. Such “complex” deletions/insertions are not rare (7387 of such variants in clinvar, accessed 2022/07/03) [26] and often lack decent tools to be correctly assessed. Thanks to SpliceAI-visual, their splicing outcome can now be predicted. Similarly, the analysis of very large size variants, like Copy Number Variants, Inversions, and Mobile Element Insertion, can be achieved with SpliceAI-visual’s Colab version. The only size limitation would be the limits of the transcript.

Taken together, although SpliceAI’s numerical DS are convenient for batch filtering, and powerful in many cases, we expose here some limitations when it comes to the careful examination of a variation in human pathology. We show the advantages of the SpliceAI-visual graphical output, RS approach to interpret splice-altering candidate variants, and we believe both tools to be complementary in the daily practice of medical genetics.

## Supplementary Information

**Additional file 1**. Supplementary Methods and Patients, contains: Patients clinical description, supplemental laboratory methods, Supplemental Figure 1, Supplemental Figure 2, Supplemental table 1.

## Data Availability

SpliceAI-visual is freely available on MobiDetails at https://mobidetails.iurc.montp.inserm.fr/MD/, or in Google Colaboratory at https://tinyurl.com/spliceai-visual. It can be freely copied for local usage, or used online in Google Colaboratory with the requirement of a Google account. All variants described in this manuscript are available in MobiDetails (https://mobidetails.iurc.montp.inserm.fr/MD/auth/variant_list/spliceAI_visual_2022 or https://tinyurl.com/bpyz9x6j). Variants included in the Additional file 1: Table S1 are also available in MobiDetails (https://mobidetails.iurc.montp.inserm.fr/MD/auth/variant_list/spliceAI_visual_complex_2022orhttps://tinyurl.com/49nujud4). MobiDetails code is available at https://github.com/beboche/MobiDetails and the SpliceAI REST API code designed for this work at https://github.com/mobidic/spliceai.

## Abbreviations

ACMG: American College of Medical Genetics and Genomics
AG: Acceptor gain
AL: Acceptor loss
DG: Delta gain
DL: Delta loss
DS: Delta score
BAM: Binary Alignment Map
OMIM: Online Mendelian Inheritance in Man
RS: Raw score
RT-PCR: Reverse transcription-polymerase chain reaction
PVS1: ACMG evidence of Pathogenicity with Very Strong weight-1
