# Supplementary material for: SpliceAI-visual: a free online tool to improve SpliceAI splicing variant interpretation
Source: Hum Genomics. 2023 Feb 10;17:7. doi: 10.1186/s40246-023-00451-1 (PMC9912651; doi:10.1186/s40246-023-00451-1)
Supplement: Supplementary file 1 — Additional file 1. Supplementary Methods and Patients, contains: Patients clinical description, supplemental laboratory methods, Supplemental Figure 1, Supplemental Figure 2, Supplemental table 1. [file 40246_2023_451_MOESM1_ESM.docx]

Supplementary Methods and Patients

### **Patients**

Patient 1 (de novo *SETD5* case) was the first child of healthy, non-consanguineous parents with no significant family history. The patient was born at 37 weeks gestation after an uneventful pregnancy with normal weight of 2750g (-1.5 SD), height of 46cm (-1.7 SD) and head circumference of 35 cm (+1 SD). A ventricular septal defect (VSD) was diagnosed at the age of 6 months which required surgery at 9 months of age. Developmental milestones were significantly delayed. She could walk independently at the age of 24 months. First words were pronounced around the age of 2 years and a half, and first association of words at 3 years. At 5 years of age, she could make simple sentences with pronunciation difficulties. She showed moderate learning difficulties and limited attention span. An orientation to a specialized school was decided after primary school. Ear tubes were placed at the age of 5 years and a half because of recurrent otitis media. She showed some difficulties in falling asleep and experienced nocturnal awakenings, treated by melatonin. At the age of 8 years and a half, the patient’s weight was 31 kgs (+1.5 SD), and height was 128.5 cm (+0.5 SD). Morphological examination revealed mild dysmorphic features: synophris, hypertelorism and hypertrichosis. Molecular karyotyping revealed a 735 kb gain on chromosome 15 (15q25.1) inherited from her asymptomatic father, which was not in favor of pathogenicity.

Patient 2 (*GRN* case) was a 70 year-old male with Fronto-Temporal Dementia (FTD), addressed to the Memory Center for a cognitive evaluation. Since his retirement, he showed behavioral abnormalities (disinhibition and exhibitionism). Following hospitalization for angioedema and the administration of systemic corticoids, he developed other behavioral changes with unrest and verbal stereotypies. His family history included Alzheimer's disease in his mother (who died at 98 years of age) and memory problems in his maternal grandfather, who died at 75 years. An initial assessment revealed a behavioral dysexecutive syndrome associated with significant impairment of social cognition and mental flexibility compatible with frontal damage. The results of the cerebral PET were more evocative of a bipolar disease rather than FTD. The patient was first treated with lithium, which was changed for valproic acid due to poor tolerance. Another assessment was then performed; memory problems such as frequent forgetfulness and misplacing of objects were noted in connection with attention problems. The neurological examination found an extrapyramidal hypertonia with a bilateral tremor and a stare. Brain MRI showed diffuse cortico-subcortical atrophy predominant in the frontal regions accompanied by hippocampal atrophy. Two plasma assays showed a decreased levels of progranulin (PGRN, produced from *GRN*) similar to those of individuals with monoallelic alteration of *GRN* (48 and 54 µg/L, normal values > 72µg/L).

Patient 3 (*CASK* case) was the first child of healthy non-consanguineous parents. His mother also had two healthy daughters from a first union. His mother had four half-sisters and two half-brothers on her mother’s side. One half-brother presented with learning disabilities and one half-sister presented with intellectual disability (Fig. 4). One niece, from one other half-sister, also had intellectual disability and was in a specialized school. For patient 3, pregnancy was marked by an oligoamnios and a fetal bradycardia at the end. He was born full term, with normal measurements: weight 3310g, height 48 cm, head circumference 34.5 cm. Independent walking was achieved within the normal range (14 months). Language development was significantly delayed. He showed psychomotor agitation, which was treated by a neuroleptic (Risperidone) since the age of 11 years. There was no feeding or sleep disturbance. Patient 3 was admitted to a specialized school after primary school. Reading and writing were not acquired at 13 years of age . Psychometric evaluation showed heterogeneous results: Verbal Comprehension Index was within the normal range (98), but other indices were low (between 59 and 67 years). Cerebral MRI performed at 9 years of agewas normal. At the age of 9 years and 7 months, he had microcephaly (OFC 49 cm, -3 SD), with normal height 133cm (0 SD) and weight 30.7 kgs (+0.5 SD). There were no dysmorphic features. Molecular karyotyping showed a heterozygous gain in chromosome 6 (6q16.3) of 1.7 Mb, which contained no OMIM genes and was inherited from his asymptomatic father, which was not in favor of its pathogenicity. Fragile-X study was negative.

Patient 4 (*TTN* case) was 39 years of age, without family history. He was referred for  elevated creatine kinase levels of around 3000-4000 IU/L, with moderate fatigability of the lower limbs. The walking perimeter remained unlimited, and the patient was still running and doing sports. The MRI showed impairment of various muscles including the semimembranosus, soleus, internal and external gastrocnemius muscles. The muscle biopsy showed numerous nuclear internalizations.

Patient 5 (inherited *SETD5* case) was a 6 year-old child presenting with a neurodevelopmental disorder associating language development and learning disabilities. He had a large stature associated with synophris, bilateral epicanthus and protruding ears. He went to a normal school, but with a personalized assistant. CGH array, trio exome sequencing and *FMR1* CGG amplification found no abnormality.

### **Genome sequencing of the *SETD5* cases (patient 1 and 5)**

Trio-based genome sequencing of both *SETD5* cases were performed at the Laboratoire de Biologie Médicale Multi Sites SeqOIA (https://laboratoire-seqoia.fr/). DNA was extracted from blood cells, and fragmented using sonication (LE220plus®, Covaris®). Libraries were prepared using the PCR-free protocol NEBNext® Ultra II End repair/A-tailing module & Ligation module, New England Biolabs®. Paired-end (2x150) sequencing was performed on Flow Cell S4, NovaSeq 6000®, Illumina®. Raw output was demultiplexed (bcl2fastq®, v2.20.0.422, Illumina®) and aligned on the GRCh38 reference genome using BWA-MEM, 0.7.15. Duplicates were marked using Picard MarkDuplicates (2.8.1), base quality was recalibrated with GATK4 (v4.1.6.0, Broad Institute). Small variants were called using GATK4 (v4.1.7.0, Broad Institute) and annotated by SNPeff (4.3t), SnpSift (4.3t). Structural variants were called with ClinSV (1.0) and annotated by AnnotSV (v3.0.7). An average depth-of-coverage of 50x was obtained for both probands, and variants were prioritized according to impact, frequency, and segregation.

### **Exome sequencing of the *CASK* case (patient 3)**

DNA was extracted from blood samples and we performed exome sequencing on the proband with the Twist Human core + refseq exome (Twist Bioscience), according to the manufacturer’s instructions, and we generated 75-bp paired-end reads on an Illumina NextSeq500. Fastq files were aligned to human genome hg19 with bwa mem (v0.7.3). We then called SNVs and DELINS following GATKs best practices (v3.4). We achieved an average mean target coverage of 98X. Variants were annotated using ANNOVAR and filtered with in-house scripts to keep variants with at least 9 reads and with a variant read frequency over 20 percent impacting exonic sequences or splice sites (+/- 10bp from the junction) and with an allele frequency <0.5% in 1000 genomes, genome aggregation database (gnomAD, 123,136 exomes and 15,496 whole genome sequences; accessed on 11/10/2018) and in a local database. The possible functional impact of amino-acid changes was predicted by SIFT (Sorting Intolerant from Tolerant), PolyPhen-2 hvar and CADD score (Combined Annotation Dependent Depletion). The Alamut software (Interactive biosoftware) was used to study retained variant sites. The variant in *CASK* was confirmed in the proband by Sanger sequencing and has been shown to be inherited from heterozygous mother.

### **Plasma progranulin assay**

Plasma progranulin levels were measured by ELISA using the progranulin-human-ELISA kit (Adipogen, Coger SAS, France), according to the manufacturer’s instructions. The antibody epitopes are located at the C-terminus of progranulin, after the Granulin E/7 sequence. The analytical performances of the kit have been previously studied in our laboratory, as the normal values vary depending on the different studies and laboratories, the normal values were defined as > 85µg/L.

### **Molecular screening of *GRN***

Molecular screening of *GRN* was performed on the proband’s blood sample by Sanger Sequencing, as previously described.^1^

### **RNA analysis for the *GRN* case (patient 2)**

RNA was extracted from fibroblast of the heterozygous proband, using the RNeasy plus mini kit (Qiagen, Düsseldorf, Germany). Cells were treated or not with emetine, a NMD inhibitor.

Reverse Transcription (RT) was performed using the SuperScript III first strand ® kit (Life Technologies SAS, Villebon-sur-Yvette, France), and RT-PCR was performed on cDNA with different conditions: one reaction with the forward primer in exon 1 (5’-TGGCCAATGGAAACTGAGG-3’) and the reverse primer in exon 3 (5’- GATGCCTGCTCAGTGTTGTG-3’), another with the forward primer in exon 1 (5’-TGGCCAATGGAAACTGAGG-3’) and the reverse primer in intron 1 (5’- GGGGAACCTCAAGCTCACAT-3’). The PCR products were analyzed by Genomic DNA Screen Tape assay with the Agilent 4150 TapeStation (Agilent Technologies).

The amplified fragments were sequenced on an ABI 3730 automated sequencer using the Big Dye 3.1 cycle sequencing kit (Applied Biosystems, Foster City, CA). The sequencing data were analyzed using SeqScape 3 software (Applied Biosystems, Foster City, CA).

RNA sequencing was performed using the mRNA stranded kit (Illumina, San Diego, CA, USA).

Strand-specific sequencing libraries were prepared using Illumina Stranded mRNA Prep. Paired-end 75-bp sequencing was performed on NextSeq 500 Illumina platform, demultiplexing and raw sequences were obtained using Illumina’s bcl2fastq. Reads were mapped to the GRCh38 human reference genome using STAR software (Dobin et al., 2013) and analyzed using Integrative Genomics Viewer (IGV) (Thorvaldsdóttir et al., 2013). Global quality was assessed using FastQC, RNA-SeQC, Picard Tools and MultiQC.

### **RNA analysis for the *SETD5* and *CASK* cases (patients 1, 3 and 5)**

Whole blood was collected in a PAXgene (PreAnalytiX) blood RNA tube and total RNA was extracted using the PAXgene blood RNA kit according to the manufacturer’s instructions (PreAnalytix). Reverse Transcription (RT) was performed with the ProtoScript II First Strand cDNA Synthesis Kit (New England Biolabs) and RT-PCR was performed on cDNA using Platinum DNA Polymerase from (Thermo Fisher Scientific). Primer sequences and conditions for PCR are available upon request. The PCR products were analyzed by DNA Screen Tape assay with the Agilent 4200 TapeStation (Agilent Technologies). The amplified fragments were sequenced on an ABI 3500xl automated sequencer using the Big Dye Terminator v1.1 cycle sequencing kit (Applied Biosystems).

For RNA sequencing, libraries were performed on Magnis NGS Prep System using the SureSelectXT HS mRNA procedure adapted for SureSelect Human All Exon V8 targeted enrichment (Agilent Technologies). Libraries were then sequenced on NextSeq 500 Illumina platform according to the paired-end 75-bp strategy (Illumina). Fastq files were aligned on GRCh38 using STAR (v.2.5.2b). BAM files were visualized with Integrative Genomics Viewer.

### **RNA analysis for the *TTN* case (patient 4)**

mRNAs extraction from a muscle biopsy and RNAseq experiments were performed by poly-A selection of mRNA, as reported in Perrin, A. *et al.*^2^

Supplemental Figure 1


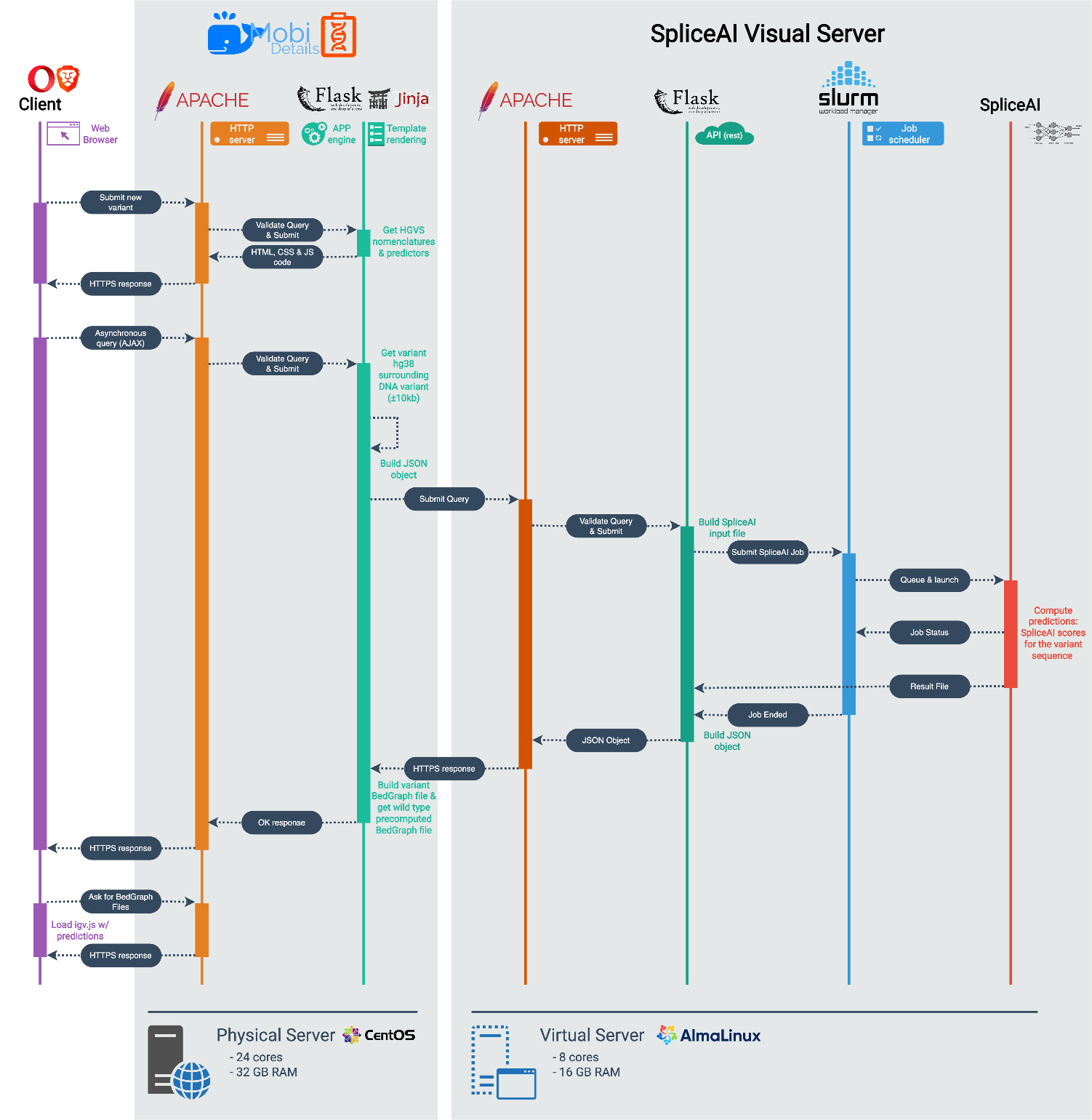


Supplemental Figure 1*: Sequence diagram of SpliceAI-visual implementation in MobiDetails.*

*MobiDetails is an online web tool available via any modern web browser. Users can submit any small variant (< 50bp) located within a human gene (19,068 genes currently available). MobiDetails returns as a first step aggregated data such as population frequencies and missense and splicing predictions (when available). When the main web page is loaded in the client web browser, an asynchronous query is sent to the web server to compute the spliceAI scores, in order to display the SpliceAI-visual results.*

*Wild-type raw scores (RS) are already pre-computed for each nucleotide of the transcripts (50,271 transcripts pre-computed). The challenge of the query is to compute SpliceAI RS for the variant sequence. To achieve this goal, the main server sends to the SpliceAI private server the variant sequence (surrounded by 10 kb of DNA sequence), embedded in a JSON object. This sequence is computed by the SpliceAI software (v1.3.1) using Illumina® models available for non-commercial usage (see https://github.com/Illumina/SpliceAI for more details). The SpliceAI RS are then embedded in a JSON object and returned to the main server. The latter builds the corresponding bedGraph file and sends to the client web browser the signal to load an igv.js genome browser as well as the wild-type and the mutant bedGraph files, allowing the user to perform the SpliceAI-visual analysis.*

Supplemental Figure 2


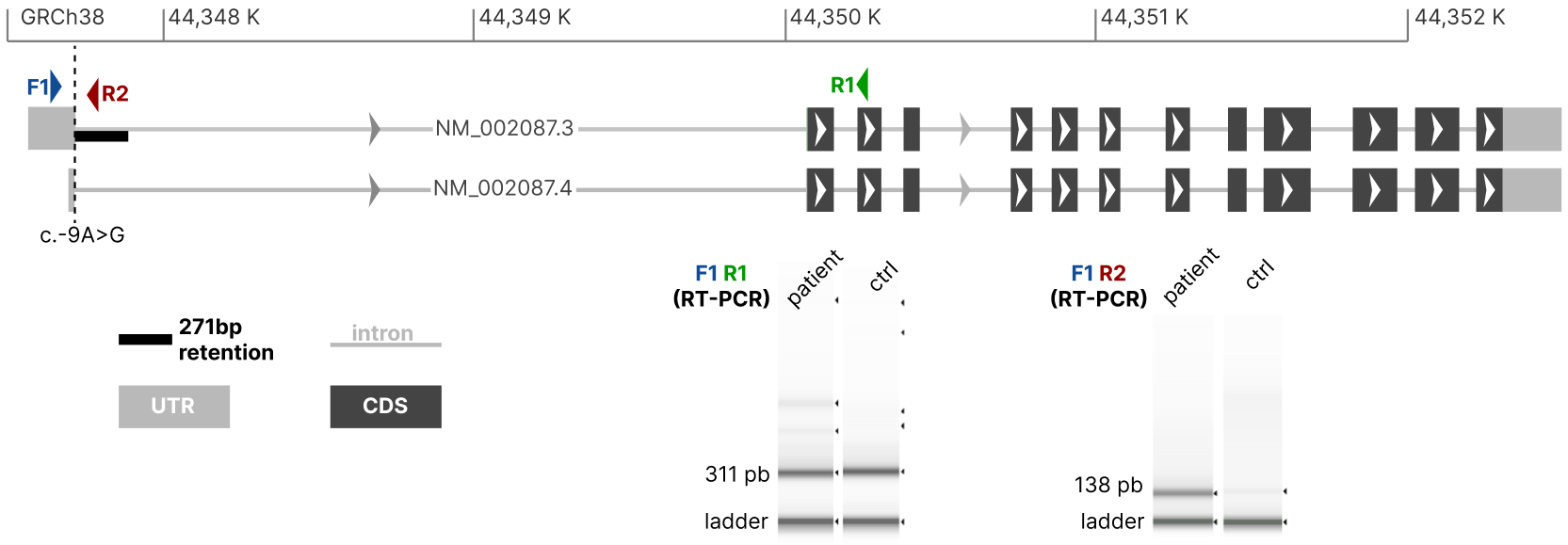


Supplemental Figure 2*: Repositioning the reverse primer for GRN transcript analysis. Above, position of the primers used in the two consecutive Reverse Transcriptase PCR (RT-PCR). Below, migration of RT-PCR products on TapeStation, showing the initial absence of any significant abnormal products (left) and the successful second result (right) with the new reverse primer (R2), showing the abnormal amplification only in the heterozygous patient, and not in the control.*

Supplemental table 1

| **complex delins** | **Alteration predicted?** | **PMID** |
| --- | --- | --- |
| NM_000051.4(*ATM*):c.4956_4957delinsTT | Yes | 22006793 |
| NM_000059.4(*BRCA2*):c.8954-1_8955delinsAA | Yes | 22632462 |
| NM_002197.3(*ACO1*):c.267-1_267delinsTA | Yes | 29534684 |
| NM_006186.4(*NR4A2*):c.865_866delinsACTCCTTTTT | Yes | 32366965 |
| NM_000492.4(*CFTR*):c.4242_4242+1delinsTT | Yes | 29750258 |
| NM_006208.3(*ENPP1*):c.2230+1_2230+3delinsCACC | Yes | 21745613 |
| NM_001368894.2(*PAX6*):c.10+3_10+4delinsCG | Yes | 9138149 |
| NM_004937.3(*CTNS*):c.225+5_225+6delinsCC | Yes | 11708862 |
| NM_000527.5(*LDLR*):c.1359-31_1359-23delinsCGGCT | Yes | 8872473 20929868 |
| NM_000059.4(*BRCA2*):c.9118-4_9118-2delinsTTT | Yes | 29750258 |
| NM_031443.4(*CCM2*):c.205-2_205-1delinsT | Yes | 18300272 20929868 |
| NM_000249.4(*MLH1*):c.1667+2_1667+8delinsATTT | Yes | 9245993 |
| NM_000202.8(*IDS*):c.1006+1delinsTT | Yes | 8281149 20929868 |

Supplementary Table 1: 13 functionally attested deleterious complex delins, collated in Li et al.^3^ All these variants and the associated SpliceAI-visual predictions can be retrieved with the following URLs:

<https://mobidetails.iurc.montp.inserm.fr/MD/auth/variant_list/spliceAI_visual_complex_2022> or

<https://tinyurl.com/49nujud4>

References

1. Le Ber I, Camuzat A, Hannequin D, et al. Phenotype variability in progranulin mutation carriers: a clinical, neuropsychological, imaging and genetic study. *Brain*. 2008;131(3):732-746. doi:10.1093/brain/awn012

2. Perrin A, Juntas Morales R, Rivier F, et al. The importance of an integrated genotype-phenotype strategy to unravel the molecular bases of titinopathies. *Neuromuscular Disorders*. 2020;30(11):877-887. doi:10.1016/j.nmd.2020.09.032

3. Li K, Luo T, Zhu Y, et al. Performance evaluation of differential splicing analysis methods and splicing analytics platform construction. *Nucleic Acids Res*. 2022;50(16):9115-9126. doi:10.1093/nar/gkac686
